# Genomewide Stabilization of mRNA during a “Feast-to-Famine” Growth Transition in Escherichia coli

**DOI:** 10.1128/mSphere.00276-20

**Published:** 2020-05-20

**Authors:** Manon Morin, Brice Enjalbert, Delphine Ropers, Laurence Girbal, Muriel Cocaign-Bousquet

**Affiliations:** a Toulouse Biotechnology Institute (TBI), Université de Toulouse, CNRS, INRA, INSA, Toulouse, France; b Université Grenoble Alpes, Inria, Grenoble, France; University of Iowa

**Keywords:** mRNA stability, *Escherichia coli*, carbon starvation, metabolic transition, posttranscriptional regulation, transcriptomic

## Abstract

Bacteria have to continuously adjust to nutrient fluctuations from favorable to less-favorable conditions and in response to carbon starvation. The glucose-acetate transition followed by carbon starvation is representative of such carbon fluctuations observed in Escherichia coli in many environments. Regulation of gene expression through fine-tuning of mRNA pools constitutes one of the regulation levels required for such a metabolic adaptation. It results from both mRNA transcription and degradation controls. However, the contribution of transcript stability regulation in gene expression is poorly characterized. Using combined transcriptome and mRNA decay analyses, we investigated (i) how transcript stability changes in E. coli during the glucose-acetate-starvation transition and (ii) if these changes contribute to gene expression changes. Our work highlights that transcript stability increases with carbon depletion. Most of the stabilization occurs at the glucose-acetate transition when glucose is exhausted, and then stabilized mRNAs remain stable during acetate consumption and carbon starvation. Meanwhile, expression of most genes is downregulated and we observed three times less gene expression upregulation. Using control analysis theory on 375 genes, we show that most of gene expression regulation is driven by changes in transcription. Although mRNA stabilization is not the controlling phenomenon, it contributes to the emphasis or attenuation of transcriptional regulation. Moreover, upregulation of 18 genes (33% of our studied upregulated set) is governed mainly by transcript stabilization. Because these genes are associated with responses to nutrient changes and stress, this underscores a potentially important role of posttranscriptional regulation in bacterial responses to nutrient starvation.

**IMPORTANCE** The ability to rapidly respond to changing nutrients is crucial for E. coli to survive in many environments, including the gut. Reorganization of gene expression is the first step used by bacteria to adjust their metabolism accordingly. It involves fine-tuning of both transcription (transcriptional regulation) and mRNA stability (posttranscriptional regulation). While the forms of transcriptional regulation have been extensively studied, the role of mRNA stability during a metabolic switch is poorly understood. Investigating E. coli genomewide transcriptome and mRNA stability during metabolic transitions representative of the carbon source fluctuations in many environments, we have documented the role of mRNA stability in the response to nutrient changes. mRNAs are globally stabilized during carbon depletion. For a few genes, this leads directly to expression upregulation. As these genes are regulators of stress responses and metabolism, our work sheds new light on the likely importance of posttranscriptional regulations in response to environmental stress.

## Abstract (precis)

The ability to rapidly respond to changing nutrients is crucial for E. coli to survive in many environments, including the gut. Reorganization of gene expression is the first step used by bacteria to adjust their metabolism accordingly. It involves fine-tuning of both transcription (transcriptional regulation) and mRNA stability (posttranscriptional regulation). While the forms of transcriptional regulation have been extensively studied, the role of mRNA stability during a metabolic switch is poorly understood. Investigating E. coli genomewide transcriptome and mRNA stability during metabolic transitions representative of the carbon source fluctuations in many environments, we have documented the role of mRNA stability in the response to nutrient changes. mRNAs are globally stabilized during carbon depletion. For a few genes, this leads directly to expression upregulation. As these genes are regulators of stress responses and metabolism, our work sheds new light on the likely importance of posttranscriptional regulations in response to environmental stress.

## INTRODUCTION

Bacteria have to face multiple environmental changes and continuously adjust their physiological and metabolic status by regulating gene expression and reprogramming cell activity. These regulations happen at multiple cellular levels, including chromosome methylation modulations and mRNA and protein concentration modifications as well as protein activity regulation (1–4). Regulation of mRNA concentration has been shown to be a crucial parameter in cell responses to environmental changes (5). In the cell, the concentration of mRNA is determined by three main factors: transcriptional activity, mRNA degradation (or mRNA stability), and mRNA dilution during cell division. Previous work suggested that the mRNA half-life (*t*_1/2_) is much shorter than the cell average doubling time (around a few minutes compared to a couple of hours) and thus that the mRNA concentration depends mostly on transcription and mRNA stability (6–10). Therefore, any changes in mRNA concentration can be the result of changes in transcription activity or in mRNA stability or both. A lot of attention has been paid to the characterization of gene expression responses to environmental changes, but the underlying mechanisms (transcription activity and/or mRNA stability) are rarely addressed. At the level of individual mRNAs, some studies have shown that stability modifications that occur in response to fluctuations in physiological and environmental factors such as growth rate, oxygen levels, nutrient levels, or temperature directly affect expression of the associated gene(s) (11–14). Therefore, this highlights an important but nevertheless still poorly characterized role of mRNA stability control in gene expression regulation, especially at the genome-scale level.

One essential challenge that bacteria have to face in their environment is limited nutrient availability. The ability to switch from favorable to less-favorable substrates and from the presence to the absence of nutrients is crucial for their growth and survival. For example, in the gut, Escherichia coli has to perpetually adjust its metabolism in the face of strong fluctuations of carbon sources, ranging from highly favorable carbon sources (mono- and disaccharides) to less-favorable carbon sources (acetate, formate, succinate) to none (15–17). This contributes not only to the survival of E. coli in the gut but also to occupation of specific niches and exclusion of potential invaders (18, 19). More generally, the ability of E. coli to switch between carbon sources has been shown to be associated with a deep reorganization of gene expression, for instance, during glucose-lactose diauxic growth or the glucose-acetate switch (20, 21). The governing mechanisms are understudied, and the role of mRNA stability and posttranscriptional regulation in adjustment to available nutrients is poorly characterized at the scale of the whole genome. This, however, would represent a crucial piece of information for our accurate understanding and further control of bacterial metabolic abilities. Thus, we need to characterize if and how mRNA stability changes when bacteria successively utilize different carbon sources from the most to the least favorable ones and cope with carbon starvation and then to investigate how these changes are involved in the establishment of the associated gene expression reorganization.

Here, we combined genomewide investigations of E. coli transcript concentrations and of half-lives during the glucose-acetate transition followed by complete carbon depletion in order to characterize (i) mRNA stability during the transition in the level of carbon sources from available to unavailable and (ii) the contribution of changes in mRNA stability to the associated gene expression response. The glucose-acetate transition followed by starvation is the most extensively used model of glycolytic to gluconeogenic metabolic switches (21–24). Thus, using a microarray-based approach, we characterized E. coli mRNA pools as well as quantified E. coli mRNA half-life values (mRNA decay analysis) at different time points representative of the different states of carbon source availability (presence of glucose, glucose depletion, acetate consumption, and starvation). We showed that mRNA half-life significantly increases over time in parallel to carbon source changes. At the same time, we observed a global decrease in levels of mRNA pools representing global downregulation of gene expression despite mRNA stabilization. However, for a limited number of genes involved in stress responses and metabolism, we show and confirm by other techniques that an increase in the half-life of mRNAs, and thus in mRNA stabilization, is responsible for their gene expression upregulation. This highlights a potentially important role of mRNA stability changes in regulation of gene expression.

Taking the results together, our work provides for the first time a genomewide investigation of the dynamic of E. coli mRNA half-life during successive metabolic switches from a favorable carbon source (glucose) to a less-favorable source (acetate) and to carbon starvation. Additionally, it opens new perspectives on the role of posttranscriptional regulations in gene expression regulation.

## RESULTS

### mRNA half-life increases during the glucose-acetate-starvation culture.

**FIG 1:**
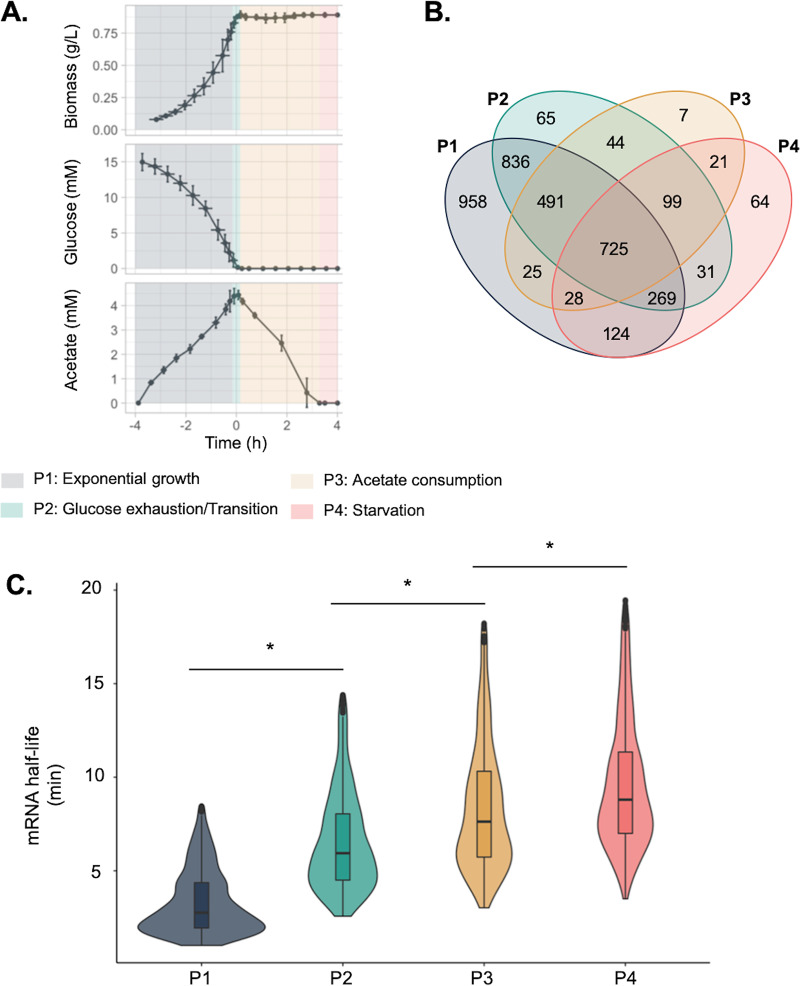
Characterization of mRNA half-life during the glucose-acetate-starvation culture. (A) Characterization of the glucose-acetate-starvation culture. E. coli was grown in bioreactors in triplicate. Biomass, extracellular glucose, and extracellular acetate levels were measured every 30 min and every 10 min with respect to glucose exhaustion. Growth and changes in levels of metabolites led to the deconstruction of the culture into the following 4 phases: P1, exponential growth; P2, glucose exhaustion; P3, acetate consumption; P4, starvation. (B) Venn diagram of mRNA with reliable half-life values identified at each time point. (C) Distribution of half-life values for the 725 mRNAs, with reliable half-life values shown for all sampled time points. The Kruskal-Wallis rank comparison test and the Dunn test for multiple pairwise comparison testing were used to compare mRNA half-life distributions. *, adjusted *P* value of <5%.

To investigate E. coli mRNA stability in glucose-acetate-starvation cultures, we carried out fermentation in triplicate in bioreactors with 16 mM glucose as the sole initial carbon source. The culture can be divided into 4 phases (phase 1 [P1] to P4) according to the nature and concentration of the carbon source (Fig. 1A). The first culture phase (P1) is characterized by E. coli exponential growth on glucose minimal media. During P1, we measured an exponential growth rate of 0.57 h^−1^ and production of 4 mM extracellular acetate (Fig. 1A; P1). Phase 2 (P2), representing the switch from a favorable to a less-favorable carbon source, corresponds to the moment when glucose is depleted, growth stops, and cells turn to acetate consumption as demonstrated previously by Enjalbert et al. (22) (Fig. 1A; P2). During phase 3 (P3), representing acetate consumption, acetate is used by E. coli as a carbon source whereas no growth (growth rate = 0) was observed, in agreement with previous work (22) (Fig. 1A; P3). Finally, phase 4 (P4), representing starvation, starts after extracellular acetate has been completely depleted and no more of the carbon source is available in the culture medium (Fig. 1A; P4).

To determine E. coli mRNA stability over time, we calculated mRNA half-life (*t*_1/2_) values for all E. coli mRNAs (4,254 mRNAs) during each phase using a microarray-based analysis. Using linear models, we calculated mRNA *t*_1/2_ values during the exponential decay of mRNA after transcription arrest (see Materials and Methods) (see Fig. S1 in the supplemental material; see also Data Set S1 in the supplemental material). Along with the *t*_1/2_ values, we calculated a coefficient of variation (cv) that allows us to assess the reliability of the half-time value (Data Set S1). To take into account potential delayed onset of the exponential decay due to transcription elongation termination after rifampin addition (10, 25), we additionally calculated mRNA *t*_1/2_ values, introducing a possible delay before the exponential decay as described previously by Moffitt et al. (25). Reliable *t*_1/2_ values calculated without delay before exponential decay correlated with a minimum *R* squared value of 0.98 (Pearson correlation) with the *t*_1/2_ values calculated with a delay (Fig. S2). We further used the *t*_1/2_ values calculated without the delay for the rest of the study. Then, we obtained reliable *t*_1/2_ values for 3,456 mRNAs during P1 (exponential growth), 2,564 during P2 (glucose exhaustion), 1,444 during P3 (acetate consumption), and 1,361 during P4 (starvation) (Fig. S3; see also Data Set S1). The large number of unreliable *t*_1/2_ values obtained after glucose exhaustion is expected to be associated with lower mRNA concentrations at those times in the culture, as 90% of the unreliable cv values were found to be associated with mRNA concentrations found within the lowest quartile of the distribution of all mRNA concentrations measured in this work.

10.1128/mSphere.00276-20.1 FIG S1 Degradation profiles of stabilized mRNAs and nonstabilized mRNAs between P1 and P2. (A) Examples of stabilized mRNAs between P2 and P1. (B) Examples of nonstabilized mRNAs between P2 and P1. Coloring is used to indicate the associated culture time point. Download FIG S1, PDF file, 1.2 MB. Copyright © 2020 Morin et al. 2020 Morin et al. This content is distributed under the terms of the Creative Commons Attribution 4.0 International license.

10.1128/mSphere.00276-20.2 FIG S2 Comparison of mRNA half-life value calculation methods. At each sampled time point, mRNA half-life values were calculated, including or not including a delay before the onset of the exponential decay. Download FIG S2, PDF file, 0.6 MB. Copyright © 2020 Morin et al. 2020 Morin et al. This content is distributed under the terms of the Creative Commons Attribution 4.0 International license.

10.1128/mSphere.00276-20.3 FIG S3 Characterization of mRNA half-life values in the glucose-acetate-starvation culture. (A) Identification of mRNA half-life values associated with reliable cv values for each culture phase. Data from mRNAs associated with a negative cv value or a cv value of greater than 40 were considered unreliable. Negative mRNA half-life values associated with negative cv values are not represented. (B) Distribution of mRNA half-life values for mRNAs with reliable half-life values at individual time points. Kruskal-Wallis rank comparison tests followed by the Dunn test for multiple-pairwise-comparison tests were used to compare mRNA half-life distributions. *, adjusted *P* value of <5%. Download FIG S3, PDF file, 0.5 MB. Copyright © 2020 Morin et al. 2020 Morin et al. This content is distributed under the terms of the Creative Commons Attribution 4.0 International license.

10.1128/mSphere.00276-20.6 DATA SET S1 Calculation of mRNA half-life values and associated coefficients of variation. This document contains a spreadsheet presenting data for each time point that reports the mRNA concentrations over time (after addition of rifampin) used to calculate mRNA half-life values. It also contains a final spreadsheet reporting the final half-life values and the associated coefficient of variation for each mRNA at each time point. Download Data Set S1, XLSX file, 4.0 MB. Copyright © 2020 Morin et al. 2020 Morin et al. This content is distributed under the terms of the Creative Commons Attribution 4.0 International license.

Comparing the sets of mRNAs with reliable *t*_1/2_ values across time points, we identified 725 mRNAs with reliable *t*_1/2_ values at any time point (Fig. 1B). While this represented only 17% of the tested mRNAs, we expected this set of genes to be unbiased and representative of what happens on the entire genome scale as no function or portion of the genome was specifically enriched. A Kruskal-Wallis rank comparison test followed by a Dunn test used for multiple pairwise comparison testing highlighted that the *t*_1/2_ distributions were different across the different culture phases, underlining that the mRNA stability changed along with the nature and concentration of the available carbon source (Fig. 1C). The median *t*_1/2_ value increased over time and was measured as 3.1 min during P1, 6.3 min during P2, 8.3 min during P3, and 9.5 min during P4, demonstrating that the mRNA half-life had increased during carbon depletion and starvation. Interestingly, the dispersion of *t*_1/2_ values toward high-stability values also increased over time, highlighting that some mRNAs can become highly stabilized during carbon starvation. Finally, we observed consistent increases and similar ranges of mRNA stability values over time in comparisons of the complete sets of reliable *t*_1/2_ values under each individual set of conditions (Fig. S3), strongly supporting the notion that the 725 genes were indeed representative of the whole genome.

Taken together, these data highlight that mRNA half-life, and thus mRNA stability, globally increases over time in correlation with changes in carbon availability from favorable to less favorable and to the absence of a carbon source.

### Significant stabilization of individual mRNAs happens at glucose exhaustion.

As the mRNA average half-life appeared to increase over the course of the experiment, we aimed to identify (i) which individual mRNAs and biological functions were significantly stabilized, (ii) when stabilization occurred, and (iii) whether stabilization lasted over time.

**FIG 2:**
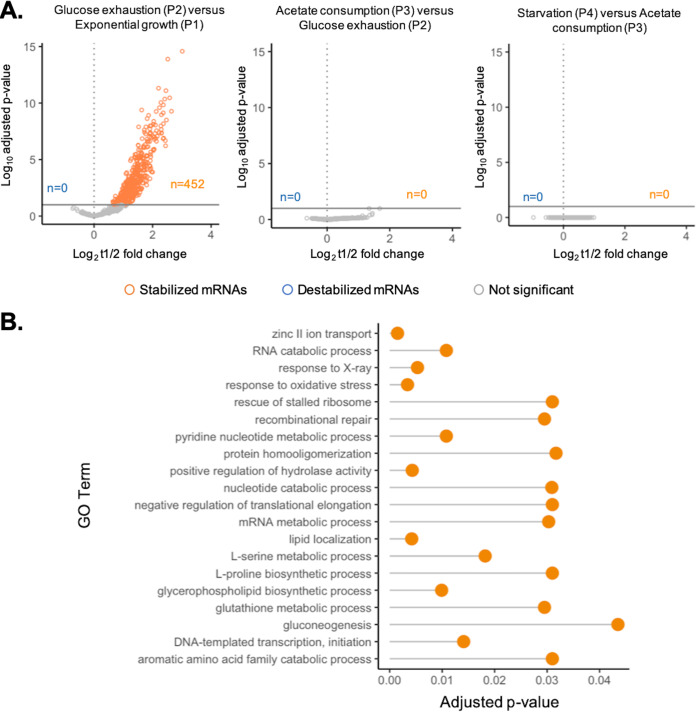
Identification of mRNAs that were significantly stabilized or destabilized between the different culture phases. The analysis has been performed on the 725 mRNAs with reliable half-life values at all sampled time points. (A) Identification of mRNAs associated with a significant fold change (FC) of half-life values. mRNAs associated with an adjusted *P* value lower than 0.1 were considered significantly destabilized (log_2_FC < 0) or stabilized (log_2_FC > 0). (B) Functional enrichment analysis of the 452 stabilized mRNAs at glucose exhaustion compared to exponential growth. Functional enrichment analysis was performed using the R package TopGO (57) as well as the E. coli annotation database org.EcK12.eg.db (58). Only GOTerm results corresponding to an adjusted *P* value lower than 0.05 were considered.

Thus, for the 725 mRNAs with reliable half-life values under all conditions, we compared *t*_1/2_ values between two consecutive time points. To statistically compare individual mRNA *t*_1/2_ values between conditions, we carried out a global model of linear regression to identify mRNAs with significant fold change *t*_1/2_ values associated with an adjusted *P* value lower than 0.1 for multiple-comparison testing. We identified 452 mRNAs whose *t*_1/2_ value significantly increased in P2 compared to P1 (stabilized mRNAs), while no mRNAs were associated with a significant decrease of *t*_1/2_ value (Fig. 2A). To validate our approach and observations, we confirmed the stabilization of a subset of five mRNAs at glucose exhaustion compared to exponential growth by reverse transcription-quantitative PCR (RT-qPCR) (Fig. S4A). The results showed that at glucose exhaustion, while no mRNAs were destabilized, the majority (62%) of the studied mRNAs were stabilized. This suggests that under our conditions, mRNA stabilization, and not mRNA destabilization, is the main form of posttranscriptional regulation involved in mRNA concentration regulation. No significant changes of individual mRNA *t*_1/2_ values were highlighted between P3 and P2 or later between P4 and P3 (Fig. 2A). This constant profile related to time after glucose exhaustion highlights that (i) no mRNA was ever significantly destabilized during the culture, (ii) the stabilized mRNAs at glucose exhaustion remained stable for the rest of the culture, and (iii) most of the stabilization occurred at glucose exhaustion.

10.1128/mSphere.00276-20.4 FIG S4 RT-qPCR validation of mRNA stabilization and upregulation under conditions of glucose exhaustion. (A) Comparison of mRNA *t*_1/2_ values between glucose exhaustion and exponential growth. *t*_1/2_ values were measured and calculated by RT-qPCR analysis of results from 8 time points after transcription arrest mediated by addition of rifampin. Experiments were performed in biological duplicate. Ratios of half-life values calculated by RT-qPCR are compared to the ratios obtained in the genomewide analysis (Microarray). (B) Differential expression analysis between glucose exhaustion and exponential growth. Analyzed mRNA concentrations correspond to the T0 values of the RT-qPCR performed for stability analysis normalized to the housekeeping gene *ihfB*. Experiments were performed in biological duplicate. Ratios of mRNA concentrations calculated by RT-qPCR are compared to the ratios obtained in the transcriptome analysis (Microarray). Download FIG S4, PDF file, 0.02 MB. Copyright © 2020 Morin et al. 2020 Morin et al. This content is distributed under the terms of the Creative Commons Attribution 4.0 International license.

We performed functional enrichment (Fisher exact test) on these 452 stabilized genes to investigate whether specific functions were significantly overrepresented. This highlighted that many stabilized mRNAs were associated with metabolic pathways, e.g., gluconeogenesis, amino acid catabolism, and nucleotide catabolism. Stabilized mRNAs were also associated with stress response (e.g., response to X-ray and response to oxidative stress) (Fig. 2B). After glucose exhaustion, cells are expected to shut down glycolytic metabolism and to reorganize their metabolism toward acetate consumption and cell maintenance in the absence of measured growth (22). Here, functions known to be involved in metabolic transition were found to be associated with stabilized mRNAs, suggesting a potential role of mRNA stabilization in the establishment of responses to carbon content and growth changes.

To conclude, our data highlight significant stabilization of most mRNAs associated with responses to exhaustion of the most favorable carbon source, indicating a potentially crucial role of mRNA stability regulation in the metabolic response. Also, these mRNAs remained stabilized afterwards, underlining that the transition from less-favorable carbon availability to the absence of a carbon source is associated with maintenance of stabilized mRNAs.

### Transcriptome analysis highlights a progressive decrease in mRNA concentrations during the glucose-acetate-starvation culture.

**FIG 3:**
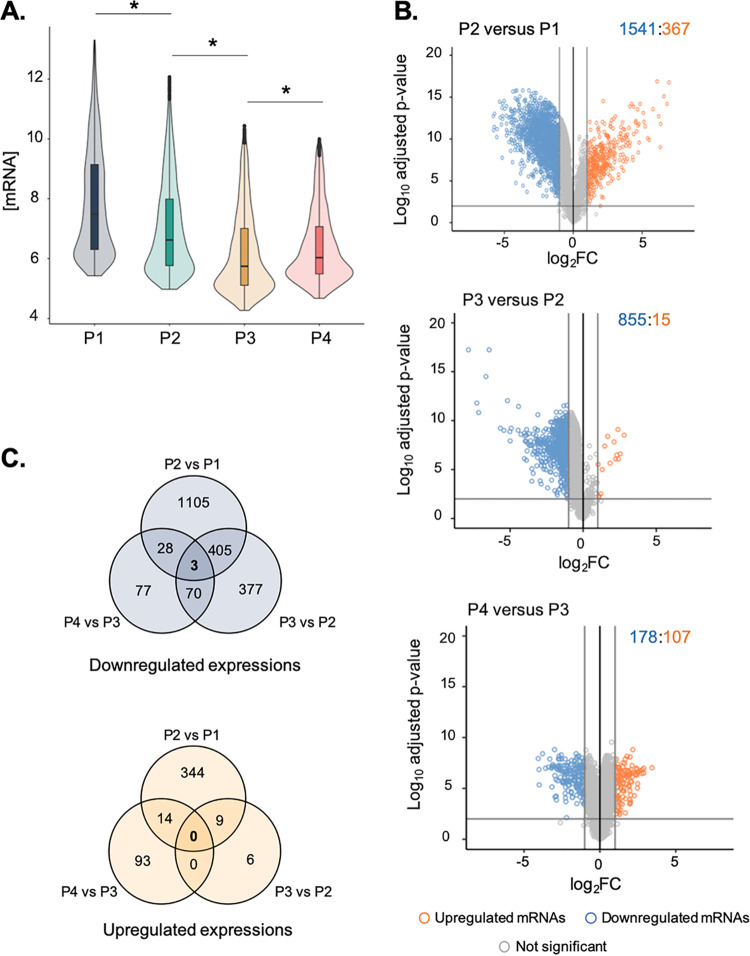
E. coli transcriptomic analysis during the glucose-acetate-starvation culture. (A) Distribution of mRNA concentrations at each time point. The Kruskal-Wallis rank comparison test and the Dunn test for multiple pairwise comparison testing were used to compare mRNA concentration distributions. *, adjusted *P* value of <5%. (B) Analysis of differential expression between consecutive culture time points. Only mRNAs associated with a log_2_ fold change value lower than −1 or greater than 1 and associated with an adjusted *P* value (Bonferroni adjustment for multiple-comparison testing) lower than 0.01 were considered differentially expressed. (C) Venn diagram of downregulated or upregulated mRNAs for each comparison.

To examine if mRNA stabilization contributed to gene expression regulation during growth of the glucose-acetate-starvation culture, we carried out transcriptomic analysis and differential expression analysis in parallel with mRNA stability analysis to identify changes in mRNA pools over time. mRNA concentrations were calculated using the microarray transcriptomic data generated for each time zero (T0) sample (before the addition of rifampin) in the stability analysis. To obtain the mRNA concentrations in arbitrary units per milligram dry weight (mgDW), intensity values were multiplied by the total extraction yield (in micrograms total RNA/mgDW) (see Materials and Methods). This highlighted a global decrease of mRNA concentration (Fig. 3A). Results from a Kruskal-Wallis rank comparison test followed by a Dunn test for multiple pairwise comparison testing highlighted that the mRNA concentration distributions were significantly different between the phases over the course of the culture.

To understand how gene expression changes with carbon source availability, we carried out differential expression analysis between two consecutive culture stages (Fig. 3B). Significant differences in mRNA concentration were determined using adjustment for multiple-comparison testing, and only mRNAs associated with an adjusted *P* value lower than 0.01 and a log of fold change greater than 1 or lower than −1 were considered to be significantly differentially expressed (see Materials and Methods). Expression levels of 1,542 genes were downregulated between P2 and P1, 855 between P3 and P2, and 178 between P4 and P3 (Fig. 3B). The number of instances of downregulated expression decreased over time, and only 3 genes were consistently downregulated between culture phases (Fig. 3C; see also Fig. S5 [circled in yellow]). The results highlight that the downregulations were culture stage specific and nonmonotonic. Taking the results together, this suggests that most downregulations happen at glucose exhaustion and that specific downregulations happen later. On the other hand, upregulation of gene expression has been detected for a lower number of genes (although three times fewer than for downregulations). Expression levels of 367 genes were upregulated between P2 and P1, 15 between P3 and P2 and 107 between P4 and P3. Upregulation of expression levels appears to have been culture stage specific but mainly occurred between P2 and P1 (Fig. 3C; see also Fig. S5).

10.1128/mSphere.00276-20.5 FIG S5 Analysis of monotonic and nonmonotonic regulation patterns over the course of the growth of the glucose-acetate-starvation culture. An upset plot (1) shows the intersections between the levels of expression of sets of upregulated and downregulated genes during the different growth phases (orange sets, upregulated sets; blue sets, downregulated sets). Upset plots are conceptually similar to Venn diagrams. The connected circles indicate which sets of gene expression data are included in the intersection, and the size of the intersection is displayed in the main bar (black bars). Download FIG S5, PDF file, 0.2 MB. Copyright © 2020 Morin et al. 2020 Morin et al. This content is distributed under the terms of the Creative Commons Attribution 4.0 International license.

The E. coli transcriptional response during the glucose-acetate transition has already been characterized under similar growth conditions (26). Our results from the comparisons of P2 versus P1 are consistent with the upregulated and downregulated expression levels and functions identified in their work. For example, we observed downregulation of many metabolic pathways and transport pathways such as glucose transport as well as upregulation of fermentation and response to starvation (Data Set S2—P2 versus P1). Interestingly, 40% of these upregulated genes (147 genes) were then downregulated between P2 and P3 and/or between P3 and P4 (circled in red; Fig. S5), suggesting that part of the upregulation response is specific to glucose exhaustion (P2) but not to acetate consumption or starvation.

10.1128/mSphere.00276-20.7 DATA SET S2 Functional enrichment analysis of upregulated and downregulated gene expression in the glucose-acetate-starvation culture. Download Data Set S2, XLSX file, 0.1 MB. Copyright © 2020 Morin et al. 2020 Morin et al. This content is distributed under the terms of the Creative Commons Attribution 4.0 International license.

In this work, however, we further characterized gene expression changes between acetate consumption and glucose exhaustion (P3 versus P2) as well as between carbon starvation and acetate consumption (P4 versus P3). Between acetate consumption and glucose exhaustion (P3 versus P2), cells appear to downregulate many metabolic pathways, including sugar and amino acid transport and metabolic processes and functions associated with cell motility (chemotaxis and bacterium-type flagellum-dependent cell motility). While only 15 gene expression levels were found to be upregulated, we observed functional enrichment associated with acetate metabolism (tricarboxylic acid cycle and succinate metabolic process) (Data Set S2; P3 versus P2). Gene expression regulation between starvation and acetate consumption (P4 versus P3) appears to be associated with upregulation of stress response (pH and hydrogen peroxide), as well as with motility regulation (bacterium-type flagellum-dependent swarming motility, regulation of single-species biofilm formation) (Data Set S2; P4 versus P3). Among these upregulated genes (70/107 genes upregulated between P4 and P3), 67% were previously downregulated (expression of 49 genes was downregulated between P2 and P1 and between P3 and P2 and that of 21 genes was downregulated between P2 and P1—circled in green; see Fig. S5). Again, this particular nonmonotonic pattern of regulation highlights specific instances of expression upregulated in starvation. Many metabolic pathways are downregulated during starvation (especially amino acid and sugar and acid transport and catabolism pathways) as well as motility (chemotaxis, bacterium-type flagellum-dependent cell motility, bacterium-type flagellum assembly). This suggests that during acetate consumption and then starvation, cells first upregulate acetate metabolism before turning most nutrient uptake and metabolic pathways off, while establishing stress response (Data Set S2; P4 versus P3).

Altogether, the transcriptome analysis highlights a global and nonmonotonic decrease of mRNA concentrations throughout the culture. Most of the gene expression regulation happened at glucose exhaustion and was mainly associated with expression downregulation.

### Some upregulated gene expression is under degradational control (stabilization), whereas most gene expression is under transcriptional control.

**FIG 4:**
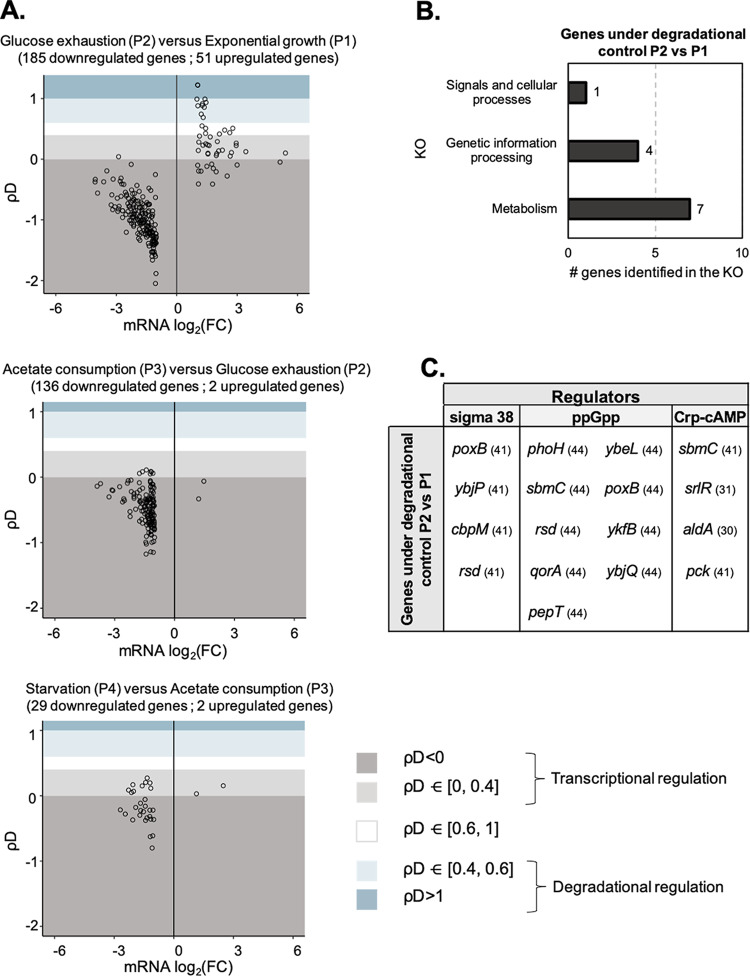
Contribution of mRNA stabilization to gene expression modification. (A) Control analysis of upregulated and downregulated gene expression levels over time. For each contrast, regulation coefficients (see Materials and Methods) associated with significant changes in expression and reliable half-life values were calculated for each comparison. (B) KEGG Orthology (KO) annotations of the 18 genes under degradational or shared control in the P2 versus P1 comparison. (C) Overlap of sigma 38, ppGpp, or Crp-cAMP regulons and genes under degradational or shared control in the P2 versus P1 comparison.

To understand if and how mRNA stabilization contributed to gene expression regulation during the glucose-acetate-starvation culture, we carried out a regulatory analysis as described previously by Esquerré et al. (7) and in Materials and Methods. This analysis calculates, for each mRNA concentration variation (between two time points), the associated degradational coefficient (ρD) and transcriptional coefficient (ρT). ρD represents the contribution of mRNA stability changes to the associated changes in mRNA concentration, while ρT represents the contribution of transcription modifications. The sum of the two coefficients equals 1, and we have defined five regulatory categories according to the ρD value (see Materials and Methods and Fig. 4). Categories 1 and 2 are associated with predominant transcriptional control (gene expression changes follow changes in transcription activity). Degradational control opposes dominant transcriptional control in category 1, while degradational control is codirectional in category 2. Category 3 is associated with equally shared codirectional transcriptional and degradational controls, while categories 4 and 5 are associated with a predominant degradational control (gene expression changes follow changes in mRNA stability).

As we aimed to understand the contribution of mRNA stabilization to gene expression regulation over time, the analysis was performed between consecutive time points. For each comparison, only the mRNAs that were differentially expressed (upregulated or downregulated) and that were part of the 725 mRNAs with reliable half-life values were analyzed. Then, regulation analysis was performed on 236 mRNAs for the comparison of glucose exhaustion time versus exponential growth (P2 versus P1), on 138 mRNAs for the comparison of acetate consumption phase versus glucose exhaustion time (P3 versus P2), and on 31 mRNAs for the comparison of starvation phase versus acetate consumption phase (P4 versus P3) (Fig. 4A). For all the comparisons, we observed that most of the downregulated gene expression levels were associated with dominant transcriptional control (with effects opposite those associated with degradational control—category 1 (dark gray; Fig. 4A). Thus, for these genes, we can conclude that during the glucose-acetate-starvation culture, gene expression downregulation mainly resulted from a decrease in transcription despite mRNA stabilization. Considering the control of upregulated gene expression, 51 mRNAs met the criteria for the P2 versus P1 comparison, while only 2 mRNAs met the criteria for the P3 versus P2 comparison and the P4 versus P3 comparison. Focusing on the 51 mRNAs that showed upregulated expression under conditions of glucose exhaustion, the regulation results were more diverse than the downregulation results, as we observed mRNAs associated with each control category (Fig. 4A). A total of 33 mRNA upregulations were associated with transcriptional control (categories 1 and 2; dark and light gray; Fig. 4A), highlighting that upregulation of expression of those genes was mainly determined by increases in transcription. For 19 of these mRNAs, this was accompanied by mRNA stabilization. On the other hand, we identified 11 genes with upregulated expression levels under conditions of predominant degradational control (categories 4 and 5; light and dark blue; Fig. 4A). Upregulation of these 11 genes is therefore mainly attributable to stabilization of their mRNA. Finally, we observed 7 upregulated mRNAs under shared control (category 3; white; Fig. 4A), suggesting that increases in mRNA stability and increases in mRNA transcription equally contribute to upregulation of these mRNA pools. Taking the data together, observation of predominant degradational control and shared control highlights a determinant role of mRNA stabilization in upregulation of expression of 18 genes at glucose exhaustion (Data Set S3). This represents 35% of the 51 genes with upregulated expression levels studied here and underlines that degradational control is involved in the upregulation of gene expression. Using RT-qPCR, we measured changes in mRNA expression level and half-life at glucose exhaustion compared to exponential growth for a subset of 5 of these 18 mRNAs and confirmed both their upregulation and their stabilization (Fig. S4).

10.1128/mSphere.00276-20.8 DATA SET S3 Control analysis of gene expression regulation between glucose exhaustion (P2) and exponential growth (P2). Download Data Set S3, XLSX file, 0.01 MB. Copyright © 2020 Morin et al. 2020 Morin et al. This content is distributed under the terms of the Creative Commons Attribution 4.0 International license.

We further investigated the functions associated with the 18 genes whose expression upregulation at glucose exhaustion was under degradational control. We investigated KEGG orthology annotations of these genes, and 12 of the 18 genes were annotated. They were associated with the following three broad KEGG orthology functions: metabolism (*pck*, *poxB*, *aldA*, *pepT*, *qorA*, *msrB*, and *nnr*), genetic information processing (*srlR*, *cbpM*, *rsd*, and *sbmC*), and signals and cellular processes (*phoH*) (Fig. 4B). Metabolic genes *pck*, *poxB*, and *aldA* encode enzymes involved in gluconeogenesis and pyruvate metabolism, *pepT* encodes the aminopeptidase PepT, while *nnr* and *msrB* encode repair proteins (the nicotinamide nucleotide repair protein Nnr and the methionine sulfoxide reductase B protein MsrB, respectively). *srlR* and *rsd* encode two transcriptional factors, the glucitol repressor SrlR (or GutR) protein and the anti-σ^70^ factor Rsd protein, while *sbmC* encodes the DNA gyrase inhibitor and *cbpA* encodes the cochaperone protein CbpA. All these genes have previously been identified as being upregulated and involved in the response to glucose exhaustion (*pck*, *poxB*, *aldA*, and *srlR* [22, 27–31]), in the stationary phase (*pepT*, *rsd*, *sbmC*, and *cbpA* [32–37]) and in the response to stress (*msrB*, *nnr*, and *sbmC* [35, 38, 39]). And most of them are known to be part of the activated regulons of the major transcriptional regulators Crp-cAMP (40–41), sigma 38 (41–42), and ppGpp (43–44) involved in the response to glucose exhaustion (Fig. 4C). These upregulated genes under degradational control are thus globally associated with the establishment of the appropriate metabolic and stress response happening at this stage of E. coli culture, underlining a potentially significant role of mRNA stabilization under conditions of glucose exhaustion.

In conclusion, modification of transcriptional activity determines most of the gene expression changes that occur when E. coli adjusts to successive conditions progressing from favorable carbon availability to less-favorable states to the absence of a carbon source. However, degradational control has been observed and mRNA stabilization has been highlighted to play a substantial role in gene expression upregulation at glucose exhaustion.

## DISCUSSION

A complex multilevel network regulates the essential adaptation of microorganisms to environmental changes. Regulation of gene expression may be one of the most extensively studied levels of control, and yet the importance of the underlying mechanism(s), whether it is modification of the transcription activity or maintenance of the stability of mRNAs, remains poorly investigated at the genome scale. The changes in mRNA stability that occur in response to various levels of availability of nutrients remain largely uncharacterized, while the changing availabilities of nutrients are expected to be involved in gene expression regulation during metabolic adaptation (45). Here, we used combined analyses of E. coli gene expression (transcriptomic analysis) and mRNA stability (mRNA half-life analysis) at the level of its entire genome to study E. coli gene expression regulation during the glucose-acetate transition followed by complete carbon depletion. We have characterized E. coli mRNA concentration and stability at four different time points of the culture, including glucose consumption, glucose exhaustion, acetate consumption, and carbon starvation, thus encompassing different environmental carbon source availability states ranging from favorable to less favorable and to the absence of a carbon source as experienced by commensal E. coli bacteria in their natural environment. Transcriptomic analysis highlighted a general decrease in mRNA concentrations over time along with changes in the carbon source nature and concentration. We showed that transcriptional reprogramming during and after acetate consumption is associated with turning off nutrient import and central carbon metabolism and possibly motility. mRNA half-life analysis demonstrated that mRNAs were globally stabilized over time, starting at a median half-life value of 3.1 min during exponential growth, and were stabilized to a median of 9.5 min during starvation whereas the strongest stabilization happened at glucose exhaustion. We further quantified the individual contributions of both transcription control (transcriptional control) and mRNA stability (degradational control) to the mRNA concentration and thus to the gene expression changes and showed that transcriptional control was the main controller of gene expression under our studied conditions whereas mRNA stabilization controlled the upregulation of a subset of genes at the time of glucose exhaustion.

In our study, mRNA stabilization mainly occurred at the time of glucose exhaustion, which coincides with growth arrest. Thus, it is unclear whether stabilization specifically happens because of glucose exhaustion and adaptation to nutrient changes or because of growth rate change, the latter being known to impact mRNA stability (6, 7, 10, 45). Here, stabilization of 195 mRNAs among the 452 mRNAs stabilized at glucose exhaustion in our data set has previously been linked to direct growth rate effects, suggesting that stabilization of these genes was mostly due to growth arrest under our conditions (7) and was unlikely to have been due to nutrient changes. However, stabilization of 57% of the mRNAs at glucose exhaustion (257 mRNAs) is likely to be independent of growth effect and associated with response to glucose exhaustion. This demonstrates that stabilization of many mRNAs is specifically related to carbon source changes and not to growth rate modifications. Additionally, recent works on Mycobacterium smegmatis suggested that mRNA stability in the cell is not determined solely by its growth status but rather by the energy metabolism status that is determined by the availability of nutrients (low-energy status being associated with stabilized mRNAs) (46), supporting our observation of mRNA stabilization in response to nutrient changes. Drops in energy levels under conditions of glucose exhaustion and acetate consumption have been shown in E. coli during the glucose acetate transition (47), and we believe that the energetic control of mRNA stability could contribute to the general mRNA stabilization observed here. However, Nouaille et al. also highlighted a physical mechanism of mRNA stabilization at low mRNA concentrations directly associated with mRNA availability (48). Because mRNA stabilization was observed concurrently with a general decrease in the mRNA concentration under most of the aforementioned stress conditions in bacteria, in budding yeasts (49), and in our work, we believe that a decrease in the mRNA concentration is also very likely an underlying mechanism of mRNA stabilization in our study.

Here, we were able to investigate the effect of mRNA stabilization on gene expression regulation only at the time of glucose exhaustion. Consequently, it is challenging to conclude if these controls are specific to the acetate switch or if they could be generalized to any metabolic adaptation. However, we have highlighted that for 18 of 51 genes (35% of our analyzed set), upregulated expression was primarily and significantly associated with degradational control and the stabilization of their mRNA, giving us an unprecedented insight in the potential role of mRNA stabilization in gene expression upregulation. These 18 genes have previously been linked to response to glucose exhaustion, alternative carbon consumption, stationary phase, and stress response.

Among the 367 genes that were upregulated at glucose exhaustion, 27% of the *rpoS* regulons (stationary-phase sigma factor σ^38^) and 28% of the Crp-cAMP regulons (catabolite repression) reported on the database Regulon DB (41) were represented, confirming that the associated global responses were likely activated at that stage of the culture. A total of 18% of the stringent response (ppGpp) activated regulons (44) were upregulated, underlining a possible activation of the stringent response at the time of glucose exhaustion as well. While we investigated only 51 upregulated genes in our control analysis, 78% of the genes under degradational control (14 of 18) were part of at least one regulon of these major transcriptional regulators. Interestingly, this raises the possibility of an intricate combination of regulatory processes related to both mRNA synthesis and degradation in the establishment of an appropriate response to glucose exhaustion and, more generally, to the response to environmental changes.

While this work draws attention to the physiological relevance of mRNA stabilization, further investigations are necessary to clarify the importance of degradational control for adaptation dynamics and the underlying mechanisms and if this information can be generalized to other metabolic transitions. Altogether, our work relies on genomewide high-throughput approaches and sheds new light on a role of mRNA stabilization and, ultimately, posttranscriptional regulation in gene expression and opens new perspectives for research investigating microbial adaptation.

## MATERIALS AND METHODS

### Strains and culture conditions.

E. coli K-12 MG1655 was grown in bioreactors (BioStat B+; Sartorius) in 1 liter of M9 minimum medium supplemented with 2.7g/liter of d-glucose (pH 7) at 37°C with airflow at 1 liter s^−1^ and stirring at 300 rpm. All cultures were inoculated at an optical density of 0.2 after a preculture was grown overnight. Parameters were set and monitored using a Multifors bioreactor system (Infors, Switzerland). pO2 was followed during the entire course of the culture, and optical density (OD) was measured every 30 min.

Rifampin (500 μg/ml) was added at an OD of 2 (exponential phase), at glucose exhaustion (identified by a sudden increase in the level of pO2), at 1 h after glucose exhaustion, or at 4 h after glucose exhaustion. Cultures were performed in biological triplicate for each time point.

### Quantification of extracellular glucose and acetate.

Sampling for extracellular metabolites was performed every 30 min after culture inoculation. Glucose and acetate were quantified by the use of an H+ high-performance liquid chromatography (HPLC) system (Agilent Technologies 1200 Series HPLC and Aminex HPX-87h column for separation of acids and sugar). The analysis was carried out at 48°C using H_2_SO_4_ 5 mM as an eluent.

### Transcriptome and mRNA stability sampling.

Samples used for transcriptome and mRNA stability analyses were collected at four time points of the culture (Fig. 1) right after addition of 500 μg/ml of rifampin, which inhibits the initiation of the transcription (50). Cells (5 mg) were harvested at T = 0, 0.5, 1, 1.5, 2, 2.5, 3, 4, 5, 6, 7, 8, 9, 10, and 11 min after rifampin addition and immediately frozen in liquid nitrogen.

### RNA extraction and microarray procedure.

RNA extraction was performed according to the TRIzol method for E. coli described previously by Esquerré et al. (7). For each condition, 12 time points were extracted (see Table 1).

**TABLE 1:** Samples used for determination of mRNA half-life values

Replicate	Samples corresponding to the indicated condition			
	Exponential growth	Glucose exhaustion	Acetate consumption	Starvation
1	T0, T0.5, T2, T5	T0, T1.5, T3, T7	T0, T0.5, T4, T7	T0, T1, T4, T11
2	T0, T1, T2.5, T3, T7	T0, T0.5, T4, T11	T0, T1.5, T3, T11	T0, T1.5, T2, T7
3	T0, T1.5, T4	T0, T1, T2, T5	T0, T1, T2, T5	T0, T0.5, T3, T5

Total RNA concentration and quality were measured using a NanoDrop spectrophotometer and an Agilent Bioanalyzer (Santa Clara, CA, USA).

After extraction, RNAs were processed on E. coli K-12 gene expression arrays (Nimblegen, Roche). We followed the same procedure as that described previously by Esquerré et al. (7). Briefly, the procedure includes synthesis of a double-stranded cDNA followed by a labeling step using a one-color DNA labeling kit. The labeled cDNA were hybridized onto E. coli K-12 gene expression arrays (Nimblegen; Roche) according to the manufacturer’s instructions. Arrays were washed and then scanned with a MS200 microarray scanner (Nimblegen; Roche). Array procedures and processing of images with DEVA 1.2.1 software were performed by the use of the GeT-Biopuces platform (http://get.genotoul.fr).

### Transcriptome analysis and differential expression analysis.

Microarray-processed T0 samples were used to produce the transcriptome data set. Transcriptome analysis was performed according to a method described previously by Esquerré et al. (7) using the R computing environment and the limma (51) and affy (52) packages. Raw probe intensities (three biological replicates for each condition of the study) were submitted to RMA (robust multiarray average)-based normalization (53). For each condition, intrareplicate normalization was performed based on the quantile normalization technique (54). A second normalization step was performed across the 12 analyzed arrays using the median values of all their common invariant probe intensities. On each array, the intensity of a transcript was then determined by the RMA-summarization procedure (53). To obtain each transcript concentration value (expressed in arbitrary units/mgDW), the intensities were multiplied by the total RNA extraction yield (in μgRNA/mgDW) calculated for each condition, with the following results: 45.2 ± 8.1 μgRNA/mgDW (exponential growth), 33.6 ± 2.7 μgRNA/mgDW (glucose exhaustion), 24.3 ± 6 μgRNA/mgDW (acetate consumption), and 27 ± 2.9 μgRNA/mgDW (starvation).

mRNA concentrations were compared by the use of a modified *t* test in conjunction with an empirical Bayes method. The Benjamini and Hochberg (BH) false-discovery rate (55) was used to correct for multiple-comparison testing and to calculate adjusted *P* values. Significant differences in mRNA concentrations between two conditions were established for genes with an adjusted *P* value lower than 1% and an absolute log of fold change value greater than 1.

### mRNA stability analysis and stability comparison analysis.

mRNA stability analysis enabled us to determine the mRNA half-life corresponding to each condition according to previously described procedures (7). For each condition, 12 arrays were used to calculate mRNA half-life values; these included 3 T0 samples and samples from 9 different time points after rifampin addition. Normalization across arrays of a given condition was performed using the median values corresponding to their common invariant probe intensities. Then, for each array, transcript intensity was calculated for each transcript by RMA summarization based on the polished median of the 32 targeting probe intensities.

To calculate mRNA half-life during the phase of exponential decay, we first performed linear regression to express ln(mRNA_int_) as a function of time for each individual transcript, where mRNA_int_ represents transcript intensity (in arbitrary units). Then, we calculated *k*, the linear regression coefficient, and its associated coefficient of variation (cv) (standard error of slope/estimation of slope) (56). *k* corresponds to the transcript degradation rate constant and was considered reliable only when the cv ϵ value was between 0 and 40. Given the following formula:

(1) $$k=\frac{\text{ln}(2)}{t_{1/2}}$$

half-life (*t*_1/2_) can directly be calculated from *k*.

Differences in half-life values between two conditions were calculated using the probability value of interaction between time and condition in a global model of linear regression. *P* values have been adjusted by the Benjamini and Hochberg false-discovery-rate method (55). mRNAs with a half-life difference associated with an adjusted *P* value lower than 10% were considered significantly different.

### Determination of regulation coefficients.

The regulation coefficients ρD and ρT were determined as described previously (7). Briefly, the difference in mRNA concentrations between two conditions can be described as a function of the differences in transcription rate *V_t_* and degradation rate *k* between the two conditions as follows:

(2) $$d\left[\text{RNA}\right]=\frac{1}{k}*dV_{t}-\frac{V_{t}}{k^{2}}*\mathrm{dk}$$

which is equivalent to

(3) $$1=\frac{d\text{ln}V_{t}}{d\text{ln}[\text{RNA}]}-\frac{d\text{ln}k}{d\text{ln}[\text{RNA}]}$$

Degradation coefficient ρD ($-\frac{d\text{ln}k}{d\text{ln}[\text{RNA}]}$) and transcription coefficient ρT ($\frac{d\text{ln}V_{t}}{d\text{ln}[\text{RNA}]}$) were calculated from equation 3. ρD represents the influence of mRNA decay on the mRNA concentration and is calculated as the negative value of the slope of the double-logarithmic plot of *k* against the mRNA concentration determined before rifampin treatment for each condition. ρT represents the importance of the transcription rate with respect to the RNA concentration. Its value is directly deduced from ρD, as their sum equals 1. ρ_D_ and ρ_T_ values for transcripts included in the 5% lowest *d*ln[RNA] or *d*ln*k* results were not calculated. Five regulatory categories have been determined according to the ρD values as follows: category 1, with ρT > 1 and ρD < 0 (mainly transcriptional control with opposite degradational control results); category 2, with 0.6 < ρT < 1 and 0 < ρD < 0.4 (mainly transcriptional control with codegradational control); category 3, with ρT and ρT ε of 0.4 and 0.6 (shared control); category 4, with 0.6 < ρD < 1 and 0 < ρT < 0.4) (mainly degradational control with cotranscriptional control); category 5, with ρD > 1 and ρT < 0 (mainly degradational control with opposite transcriptional control results).

### RT-qPCR experiments and analysis.

For selected genes, gene expression and stability were monitored by RT-qPCR. For expression analyses, a volume equivalent to 2 mg of cell dry weight was harvested by centrifugation (1 min, 13,000 × *g*) and flash-frozen in liquid nitrogen. For stability analyses, the same procedure was applied and sampling was performed at times 0, 0.33, 1, 2, 5, 7, 9, and 13 min after rifampin addition (500 μg·ml^−1^) for exponential-phase analysis and at times 0, 1, 3, 5, 7, 9, 13, and 17 min after rifampin addition for the glucose exhaustion phase. Extractions of total RNA were performed using a Qiagen RNeasy Midi extraction kit. Samples were treated with DNase to eliminate genomic DNA; the samples were subjected to reverse transcription using Super Script II reverse transcriptase (Life Technology). RT-qPCR was performed using a SYBR green-based detection protocol (Life Technology) with an ICycler real-time PCR detection system (Bio-Rad) and MyIQ software (Bio-Rad). Primers (Table 2) were designed with primer3 online freeware (http://frodo.wi.mit.edu/). *t*_1/2_ values were determined as the inverse of the slope of the threshold cycle (*C_T_*) over time.

**TABLE 2:** List of primers used for RT-qPCR experiments

Primer name	Primer sequence (5′ to 3′)
Q-pck-5’	GACGCCATCCTCAACGGTTC
Q-pck-3’	GTGTCTACGCCCGGCAGTTC
Q-qorA-5’	TCGTGTAGTCTATGCGCAGTC
Q-qorA-3’	GCTCAAAAGAAATTGCCGCAG
Q-sbmC-5’	AGCAGGAAGAGAAACGTACCG
Q-sbmC-3’	ATCTACCCACATCATCAACTGC
Q-rsd-5’	TTGATCGCTGGCTACATGTAC
Q-rsd-3’	TTTCGTTTAGCCTCATGTACG
Q-srlR-5’	CAACACCCACAAGAAAGAGC
Q-srlR-3’	ACCATCTGCAAAACGGTACTG

### Data availability.

Raw and processed data were deposited in the Gene Expression Omnibus data repository and are accessible through GEO accession no. GSE144316 (transcriptome data) and GSE144315 (stabilome data).
