# Supplementary figures and images for: Genomewide Stabilization of mRNA during a “Feast-to-Famine” Growth Transition in Escherichia coli
Source: mSphere. 2020 May 20;5(3):e00276-20. doi: 10.1128/mSphere.00276-20 (PMC7380570; doi:10.1128/mSphere.00276-20)

**Fig S1**

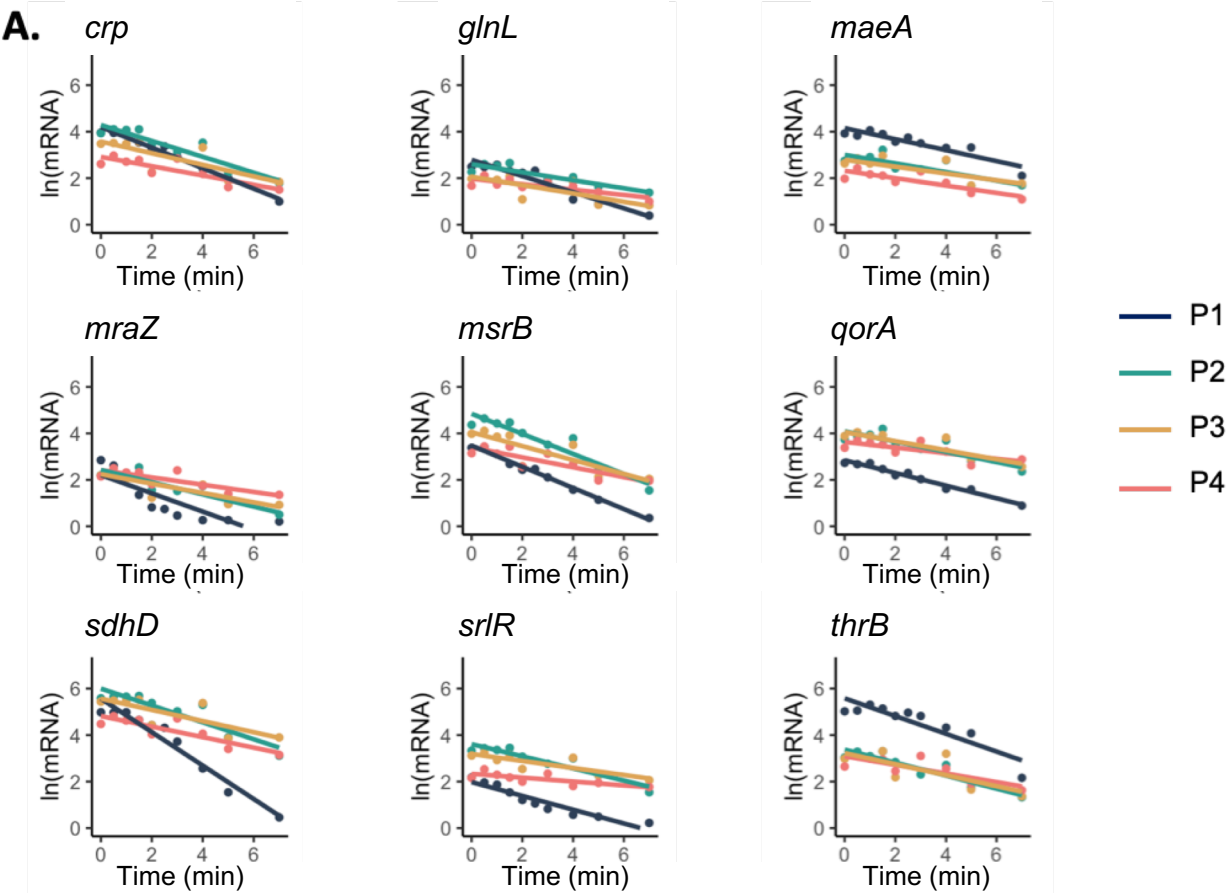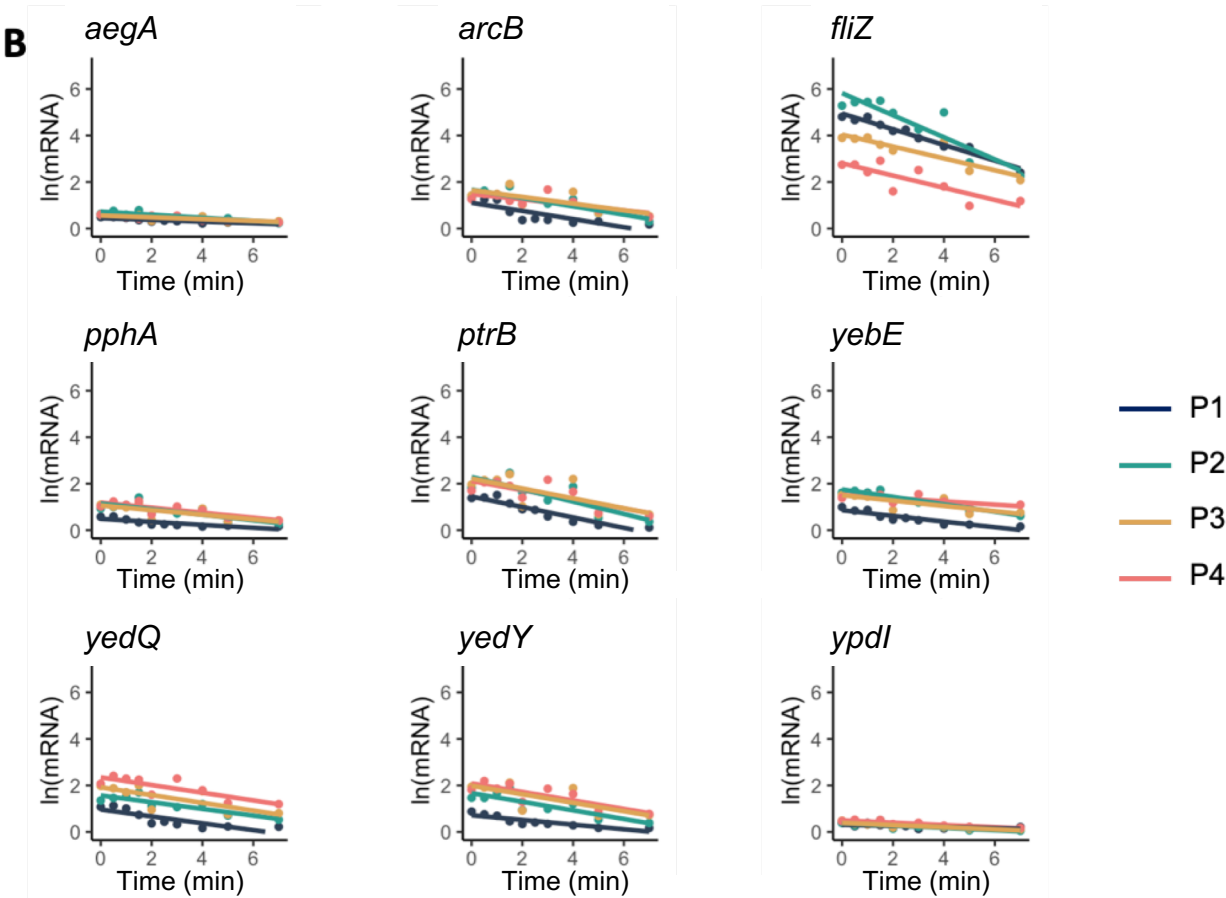

Supplement: FIG S1 [file mSphere.00276-20-sf001.pdf]

**Fig S2**

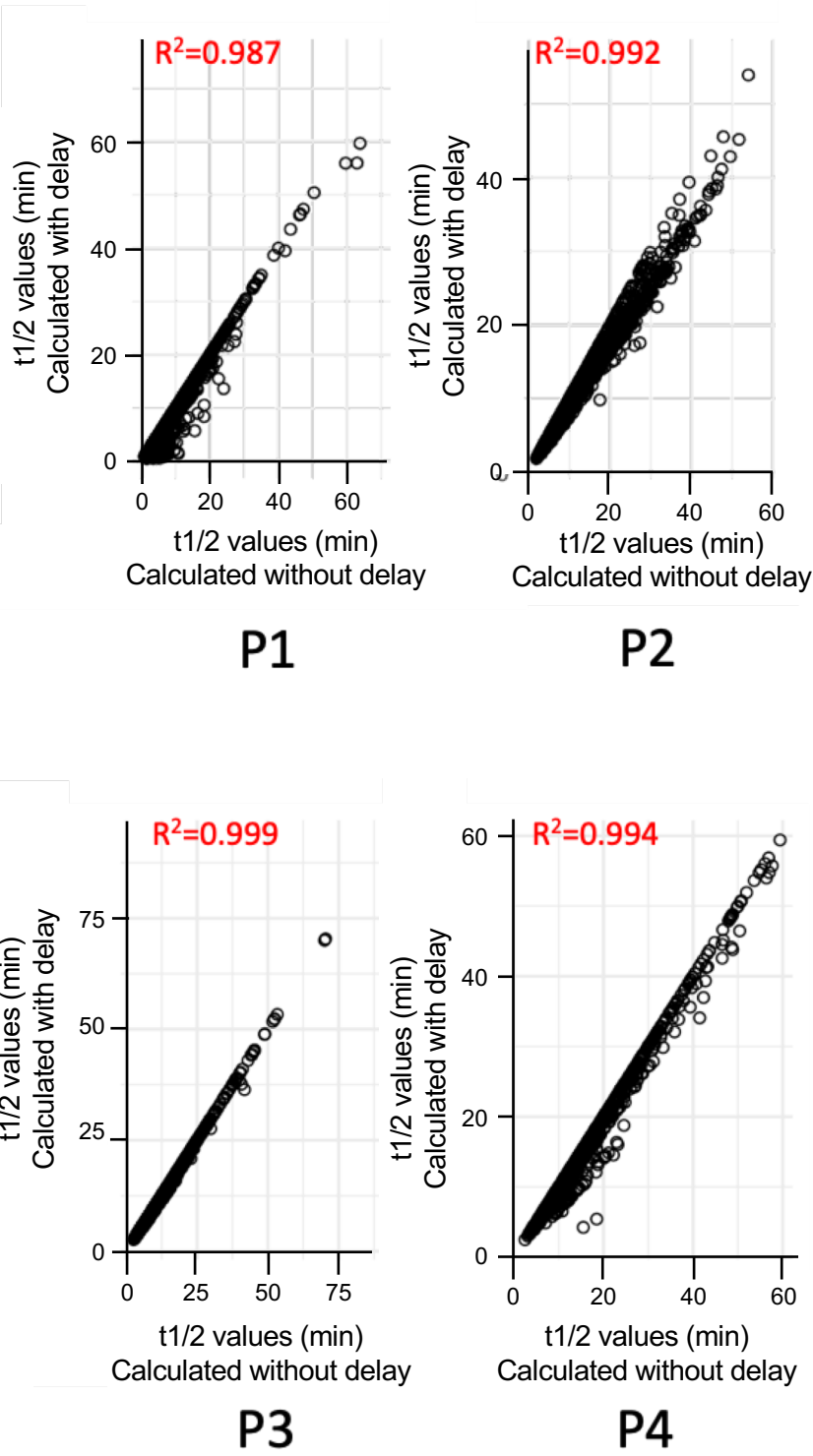

Supplement: FIG S2 [file mSphere.00276-20-sf002.pdf]

**Fig S3**

**A.**

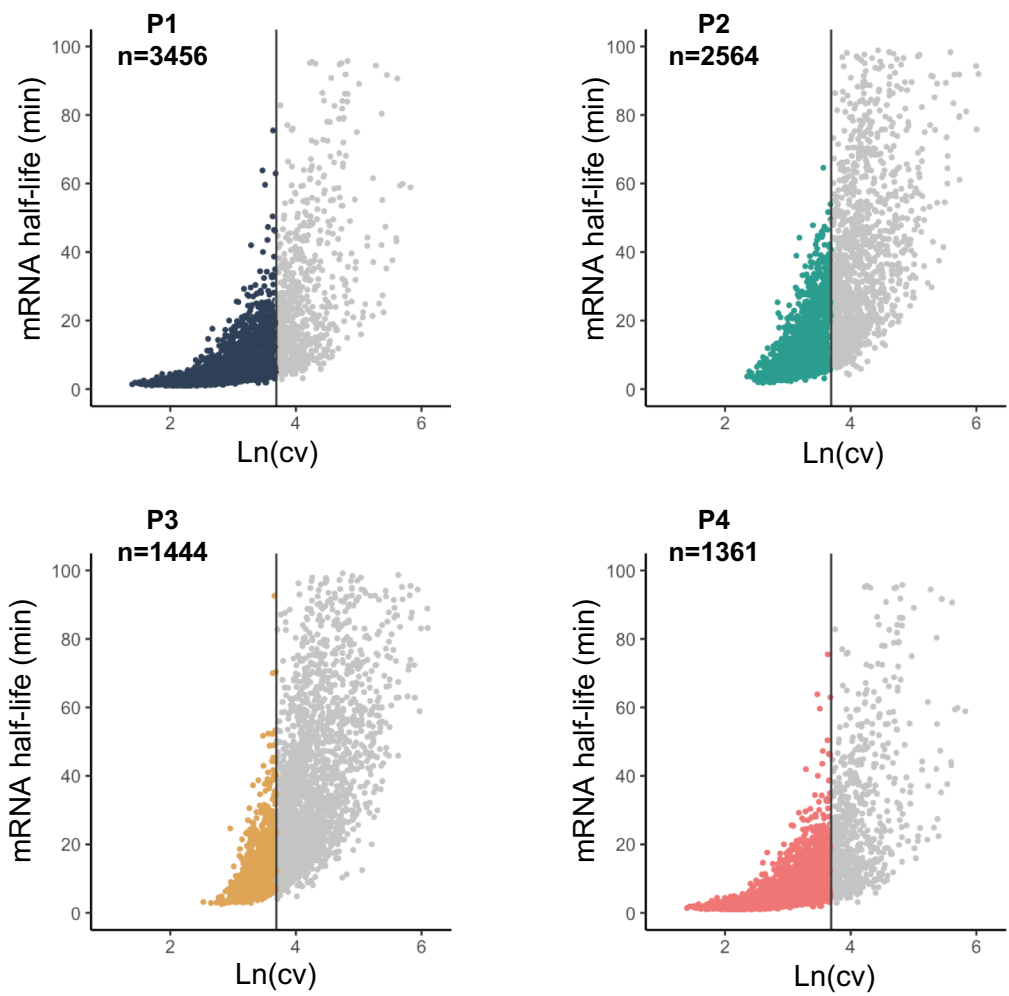

**B.**

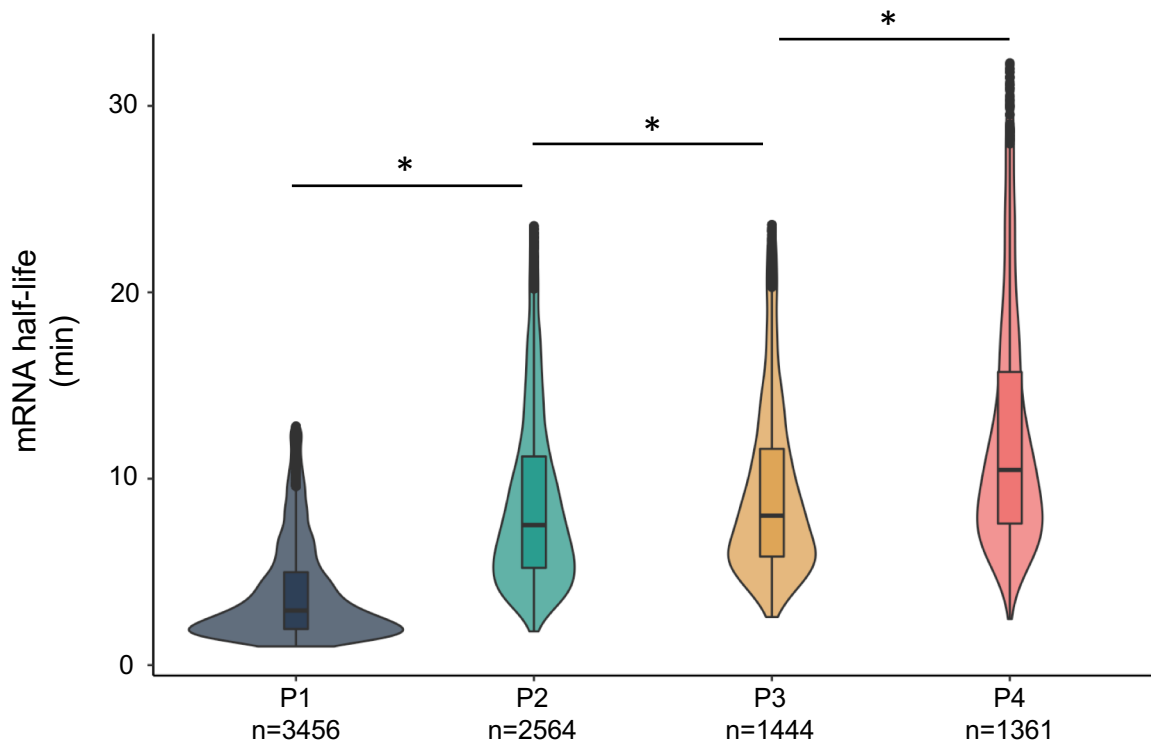

Supplement: FIG S3 [file mSphere.00276-20-sf003.pdf]

Fig S4

A.

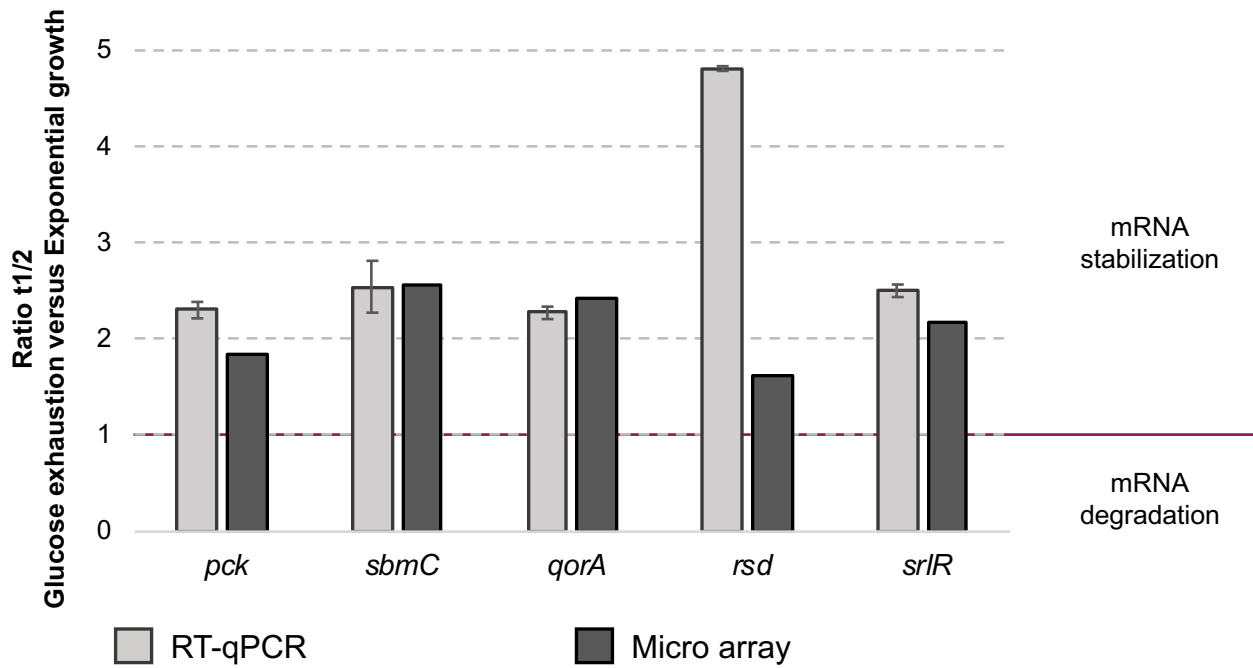

B.

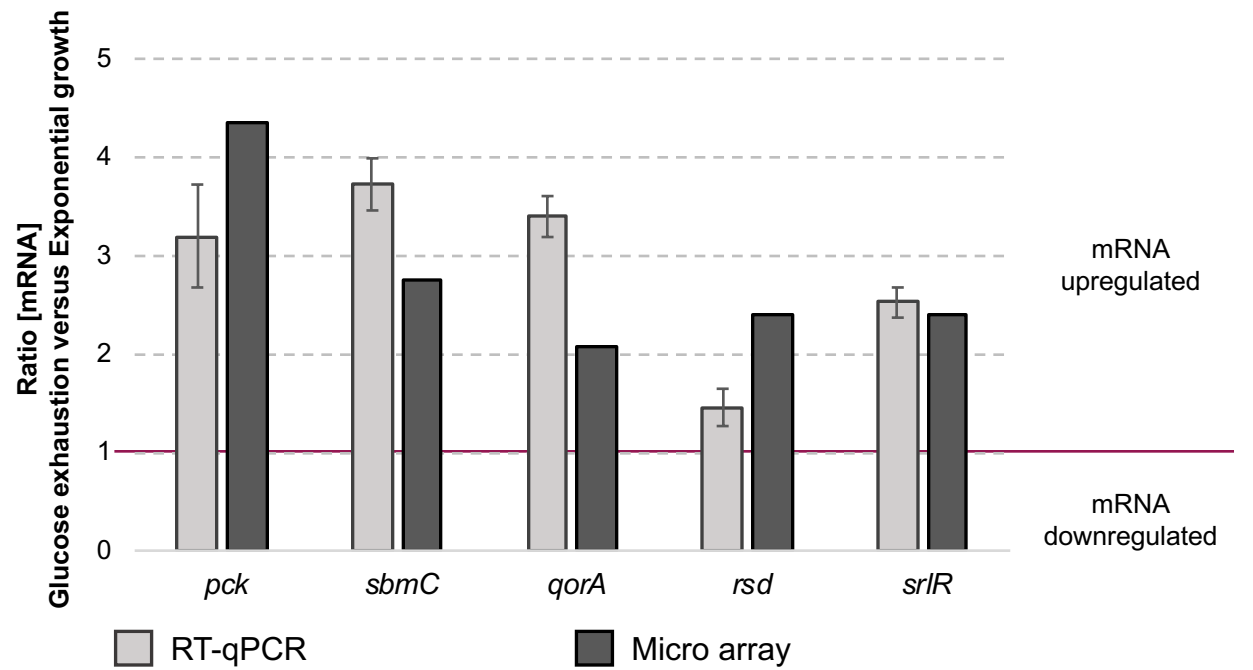

Supplement: FIG S4 [file mSphere.00276-20-sf004.pdf]

**Fig S5**

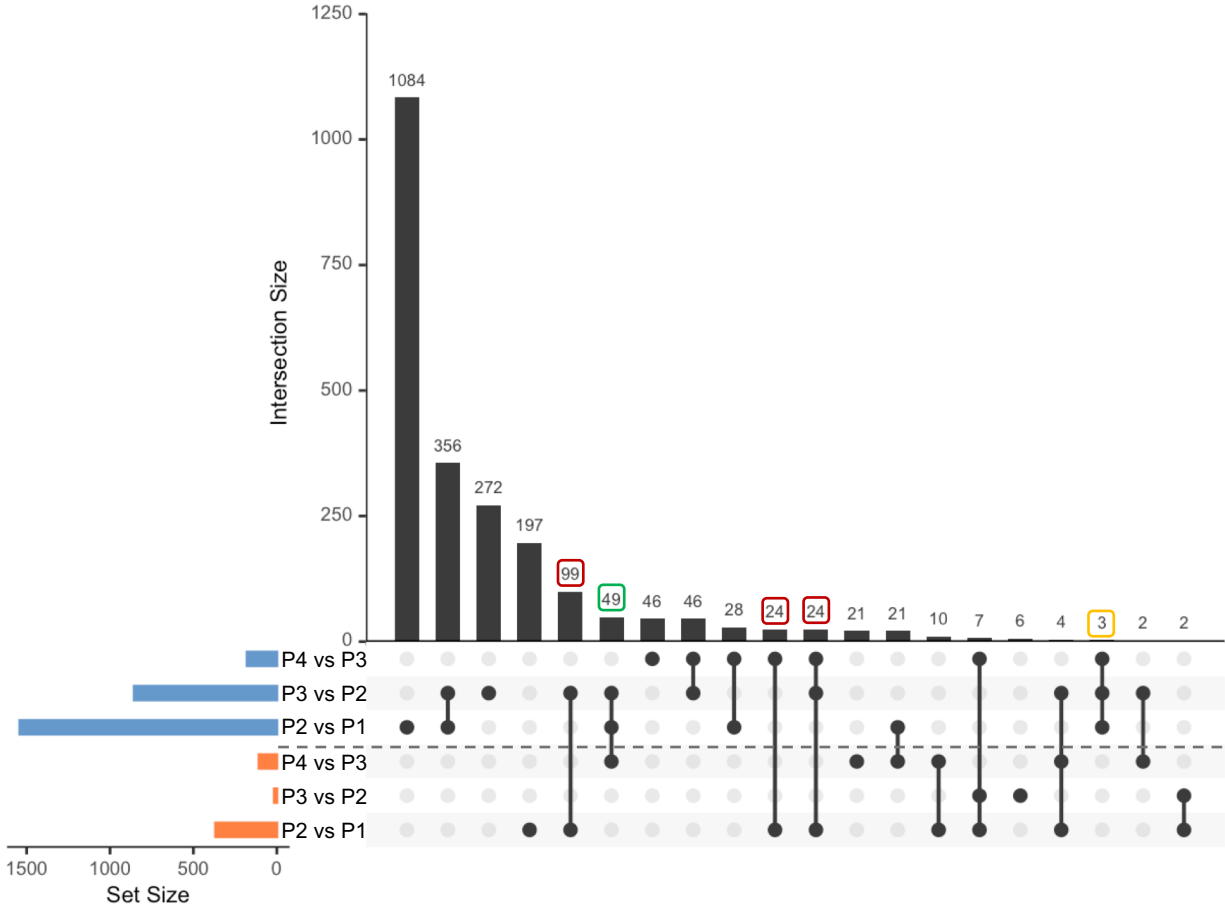

Supplement: FIG S5 [file mSphere.00276-20-sf005.pdf]
